# Transcriptome analysis of rubber biosynthesis in guayule (*Parthenium argentatum* gray)

**DOI:** 10.1186/s12870-019-1669-2

**Published:** 2019-02-12

**Authors:** Solomon H. Stonebloom, Henrik Vibe Scheller

**Affiliations:** 1 Joint BioEnergy Institute and Environmental Genomics and Systems Biology Division, Lawrence Berkeley National Laboratory, Berkeley, CA 94720 USA; 2 Department of Plant and Microbial Biology, University of California Berkeley, Berkeley, CA 94720 USA

**Keywords:** *Parthenium argentatum*, Guayule, Rubber biosynthesis, Transcriptome, Gene expression

## Abstract

**Background:**

Natural rubber is currently produced nearly exclusively from latex of the Para rubber tree, *Hevea brasiliensis*. The desire to reduce the environmental cost of rubber production, fears of pathogen susceptibility in clonal *Hevea* plantations, volatility in the price of natural rubber, and increasing labor costs have motivated efforts to diversify the supply of natural rubber by developing alternative rubber crops such as guayule (*Parthenium argentatum* Gray). In *Hevea,* latex is produced as an exudate following wounding while in guayule, rubber is deposited within the cortical parenchyma and its production is strongly influenced by environmental conditions.

**Results:**

To better understand the enzymology and regulation of guayule rubber biosynthesis and to identify genes with potential uses in the improvement of rubber yields, we conducted de novo transcriptome assembly and differential gene expression analyses of this process in guayule. This analysis supports a role for rubber in the defense against pathogens, identified new enzymes potentially involved in the biosynthesis of rubber as well as transcription factors specifically expressed in rubber-producing tissues.

**Conclusions:**

Data presented here will be useful in the improvement of guayule as an alternative source of natural rubber and in better understanding the biosynthesis of this critical polymer. In particular, some of the candidate transcription factors are likely to control the rubber biosynthesis pathway and are good targets for molecular breeding or engineering of guayule plants with higher and more consistent production of rubber.

**Electronic supplementary material:**

The online version of this article (10.1186/s12870-019-1669-2) contains supplementary material, which is available to authorized users.

## Background

Natural rubber is a critical commodity currently produced from the latex of the Para rubber tree, *Hevea brasiliensis*. Global annual natural rubber production exceeds 13 million metric tons [1]. The demand for natural rubber has grown due to economic development and increased automobile ownership in Asia. This increased demand for rubber, in addition to competition for land with more profitable oil palm cultivation is a driver of deforestation in southeast Asia and threatens biodiversity [2]. The desire to reduce the environmental cost of rubber production, fears of pathogen susceptibility in clonal *Hevea* plantations, volatility in the price of natural rubber on the commodities market, and increasing labor costs for *Hevea* rubber production motivate efforts to diversify the supply of natural rubber by developing alternative crops such as guayule (*Parthenium argentatum* Gray) and Russian dandelion (*Taraxacum kok-saghyz*). Guayule is a xerophytic perennial shrub in the Asteraceae that produces high quality natural rubber, was widely studied in the United States during World War II as part of the Emergency Rubber Project and is currently being developed as an alternative rubber crop.

Guayule has major advantages over *Hevea* for the production of rubber in that it can be cultivated broadly across temperate and arid climates. Guayule produces high molecular weight natural rubber suitable for use in the manufacture of tires. Harvesting of biomass and rubber extraction are easily mechanized, unlike the labor-intensive tapping and latex collection required for *Hevea* rubber production. In guayule, rubber is deposited throughout the stem, primarily in the cortical parenchyma. As rubber is deposited intracellularly and not in an exudate, to harvest rubber whole guayule plants are mechanically harvested, baled and then processed to chemically extract rubber and other co-products. Furthermore, guayule latex does not elicit an allergic response in individuals allergic to *Hevea* latex and extended exposure to guayule latex does not produce an allergic response [3]. Unfortunately, breeding and true domestication of guayule are complicated by its polyploidy and facultative apomictic reproduction by which approximately 90% of seeds are not the product of sexual reproduction. Naturally occurring stands and breeding populations of guayule contain individuals with ploidy levels ranging from 2n = 2x = 36 (diploid) to 2n = 5x = 90 (pentaploid) [4, 5]. Progeny of individual guayule plants frequently exhibit aneuploidy and while diploid lines exist and are currently being used in breeding programs, diploids lack vigor and have reduced biomass and rubber yields, suggesting that tetraploid lines will be necessary for cultivars entering production [4].

Unlike *Hevea* rubber production where latex exudate is produced following wounding, rubber biosynthesis in guayule occurs in cells lining the resin ducts and in the cortical parenchyma and is strongly influenced by environmental conditions [6, 7]. Rubber is made only when plants experience cold temperatures during the winter [8–10]. Several studies have characterized changes in the expression of rubber-producing genes and the activity of rubber biosynthetic enzymes following exposure of the plant to low temperatures either in the field or under controlled conditions [11, 12]. This climactic regulation of rubber biosynthesis limits the productivity of guayule plantations as rubber is produced exclusively during cold winter months. This also affects where guayule may be profitably cultivated outside of the high-desert environment of guayule’s native range. Given that there is evidence for the transcriptional regulation of rubber biosynthesis and as traditional breeding approaches to increasing the rubber content of guayule have been challenging, here we aim to identify master regulator transcription factors regulating rubber biosynthesis in guayule to enable engineering of improved lines. We hope that ectopic expression of these transcription factors throughout the year will induce constitutive rubber production, increase rubber yield and potentially expand the productive range and economic viability of this alternative rubber crop. Furthermore, differential gene expression analysis of rubber producing and control tissues will elucidate metabolic processes occurring during rubber biosynthesis and help to identify additional enzymes involved in the biosynthesis of rubber.

To this end we established conditions inductive of rubber biosynthesis in guayule and validated the induction of rubber biosynthesis by extraction and qRT-PCR of known rubber biosynthetic genes. We then conducted an RNA-seq analysis of gene expression in rubber biosynthetic and control tissues, assembled the transcriptome and performed differential gene expression analyses. As rubber is produced specifically in the stems of plants exposed to low temperatures, sequencing libraries were prepared from stem and leaf tissues of cold-treated and control plants. Genes previously suspected to be involved in rubber biosynthesis were highly expressed in rubber biosynthetic tissues. We identified a set of genes encoding transcription factors and DNA-binding proteins that were specifically expressed in rubber-producing tissue as candidate regulators of rubber biosynthesis and that may be useful for the improvement of guayule rubber yields. Analysis of gene expression in rubber-producing tissues also informs upon the biological function of rubber production and the identification of new enzymes involved in rubber biosynthesis.

## Results

### Induction of rubber biosynthesis

Rubber biosynthesis in guayule occurs when the shrub experiences low nighttime temperatures below 5–7 °C during the winter in the cortical parenchyma of stems [9–11, 13, 14]. Artificial exposure to low temperatures induces rubber accumulation in guayule in plants older than approximately 120 days [8]. Exposure to low, non-freezing night temperatures in the lab induces rubber biosynthetic activity in guayule [15]. The enzymatic rates of 3-hydroxy-3-methylglutaryl-CoA reductase and rubber transferase enzymes are also seasonally induced [16]. To study changes in gene expression associated with the production of rubber we sought to establish inductive and repressive growth conditions for guayule rubber biosynthesis in the lab and to validate that changes in the expression of genes associated with rubber biosynthesis were induced.

**Fig. 1:**
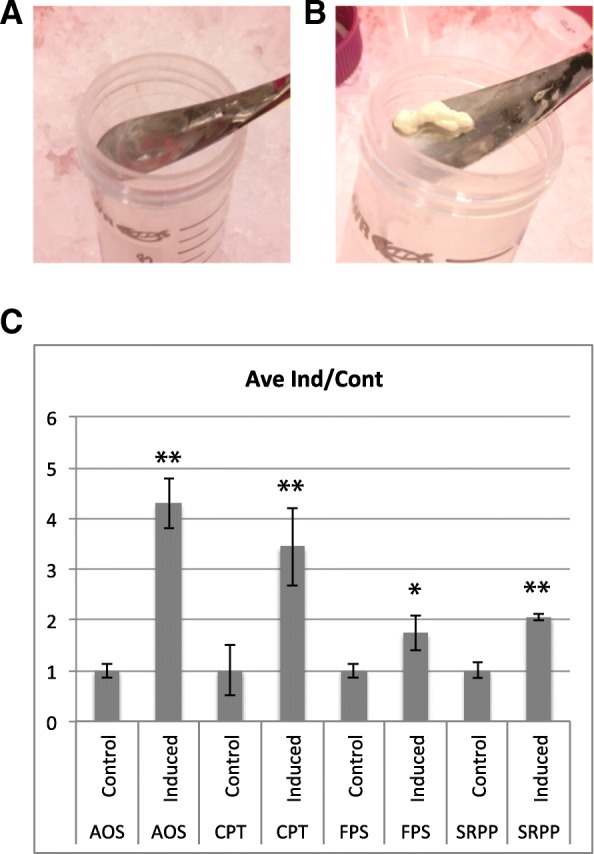
Verification of rubber biosynthesis induction in guayule plants by exposure to simulated winter conditions for 2 months. Washed rubber particle extractions from plants grown in simulated summer (**a**) and winter (**b**) conditions. **c** Quantitative RT-PCR of rubber biosynthetic enzymes (AOS, alllene oxide synthase; CPT, cis prenyl transferase; FPS, farnesyl diphosphate synthase; SRPP, small rubber particle protein). Significant differences between induced and control are shown (t-test, * *p* < 0.01, ** *p* < 0.001)

We simulated approximate summer and winter temperature regimes in guayule’s native environment with parameters in concordance with previous studies of the induction of rubber biosynthesis in guayule [11, 12]. While low temperature is likely the most important environmental factor for inducing rubber biosynthesis, we also changed daylength and day night temperature differential to simulate seasonal changes where guayule is grown. Summer conditions were simulated in growth chambers with a 16-h day with 25 °C daytime and 15 °C nighttime temperatures and winter conditions with an 11-h day with 25 °C daytime and 5 °C nighttime temperatures. To validate the induction of rubber biosynthesis we extracted rubber particles from guayule plants exposed to simulated winter and summer conditions for six weeks (Fig. 1a, b). Large amounts of rubber were present in cold-induced plants, forming a thick mat of congealed rubber following centrifugation. Little rubber was present in plants grown in simulated summer conditions. The expression levels of genes thought to be involved in the biosynthesis of rubber, AOS (Allene Oxide Synthase) and CPT (Cis Prenyl-Transferase), have been shown to be weakly correlated with the induction of rubber biosynthesis by winter conditions in guayule [12]. To validate that these genes were induced in our rubber-producing plants we tested the relative expression of previously identified genes thought to be involved in guayule rubber biosynthesis in stems of plants exposed to simulated summer and winter conditions using real-time RT-PCR. As expected, significant up-regulation of AOS, CPT and FPS (farnesyl diphosphate synthase) transcripts was detected in plants grown in simulated winter conditions (Fig. 1c). We also detected significant induction of the SRPP (small rubber particle protein) transcript, which has not previously been shown to be responsive to low temperatures. We thus concluded that simulated winter conditions were capable of inducing rubber biosynthesis in guayule and that this induction elicited changes in the expression of rubber biosynthetic enzymes.

### RNAseq analysis and transcriptome assembly

To characterize global changes in gene expression in rubber-producing tissues and to identify genes involved in the biosynthesis of rubber we performed an RNAseq analysis of control tissues (leaves) and tissues producing rubber (stems) from control and induced plants. We prepared bar-coded, directional RNA-seq libraries from the stems and leaves of 7-month-old guayule plants that had been exposed in simulated winter or summer conditions for 8 weeks (Additional file 1: Figure S1). Three biological replicate sequencing libraries were prepared from each tissue. These libraries were pooled and sequenced on MiSeq and Hiseq Illumina sequencers resulting in 28 million 300-bp paired-end reads and 155 million 150-bp paired-end reads respectively with an average of 2.4 million MiSeq and 12.9 million HiSeq reads per sample. The transcriptome was assembled using the Trinity assembler into 200,074 unigenes with a median length of 456 bp and with 50 % of the total sequence assembled into contigs of 850 bp or larger (Table 1). The total assembly represented 149 million bp of sequence. As the plants used in this study are of the AZ-2, tetraploid cultivar, the transcriptome assembly contains up to four alleles of each gene given that the plants sequenced were the progeny of a tetraploid single plant selection [5]. Many transcripts were assembled into distinct “isoforms” likely representing distinct alleles of individual genes. That the transcriptome assembly likely represents up to four distinct contigs of each gene complicates the data by adding allelic variation between individual plants to differential gene expression analysis. In the absence of a high-quality genomic sequence, it is generally not possible to distinguish between allelic variants and gene homologs. Furthermore, due to the relatively short contig length, it is obvious that several individual ‘unigenes’ can derive from the same gene locus.

**Table 1:** Summary statistics of contig assembly

Trinity ‘unigenes’	200,074
Contigs	219,958
GC content	39.90%
Contig N10	2655
Contig N20	1900
Contig N30	1451
Contig N40	1110
Contig N50	850
Median contig length	456
Average contig length	678.1
Total assembled bases	149,145,074

### Differential gene expression analysis

**Fig. 2:**
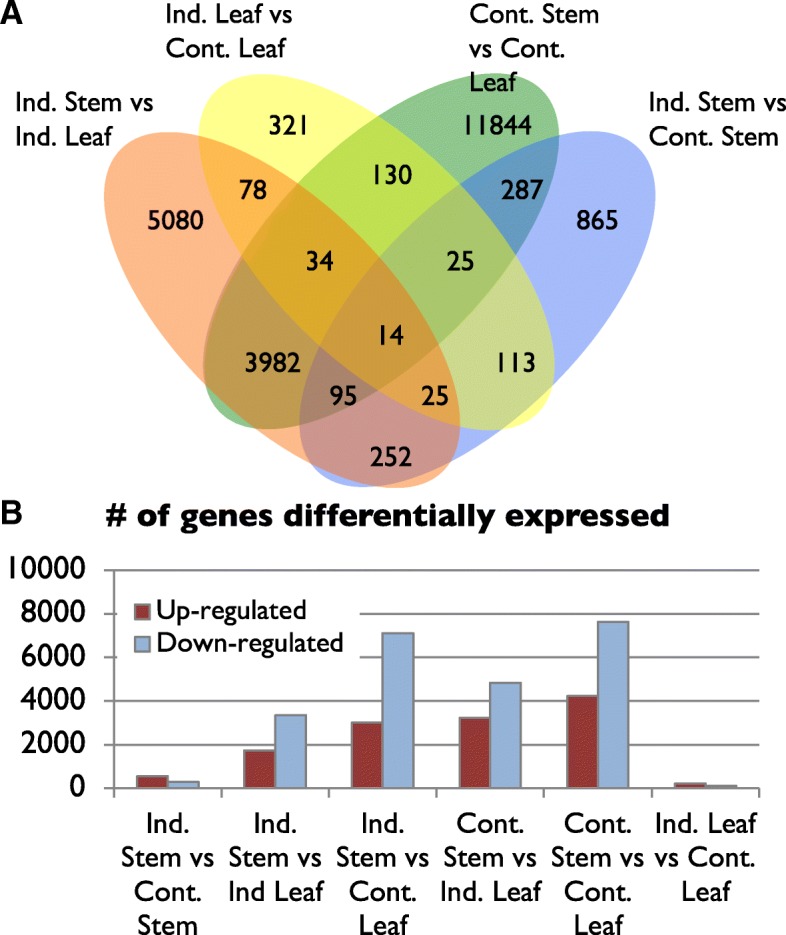
Differential gene expression in guayule transcriptome analysis. **a** Venn diagram showing the number of differentially expressed genes in common between different tissues. **b** The number of up and down-regulated genes in comparisons of gene expression between each tissue

To identify genes involved in guayule rubber biosynthesis, differential gene expression analysis was conducted using the EdgeR package [17]. Our premise was that genes involved in rubber biosynthesis would be induced, primarily by cold, under simulated winter conditions in stems, where rubber is produced, but not in leaves where it is not; whereas genes induced by cold but not related to rubber biosynthesis would be induced in both leaves and stems. 16,394 differentially expressed contigs were identified at p_adj_ < 0.0001 between two or more tissues. In a Venn diagram analysis, we analyzed the number of contigs that were differentially expressed between multiple tissues (Fig. 2a). Notably, 3982 + 34 + 14 + 95 contigs were differentially expressed between stem and leaf tissues in both induced and control plants (Fig. 2a). Only 252 contigs were differentially expressed between induced and control stems as well as between leaves and stems in induced plants, but not in induced leaves compared to control leaves, nor in control stems compared to control leaves. This set of 252 contigs represent the set where we hypothesized that genes involved in the production of rubber would most likely be identified. We also examined the number of contigs up- and down-regulated in each comparison (Fig. 2b). More genes were up-regulated than down-regulated in both stem and leaf tissues following cold treatment. Likewise, cold-induction resulted in the up-regulation of only 205 contigs in leaf tissues and the down-regulation of 116 contigs.

**Fig. 3:**
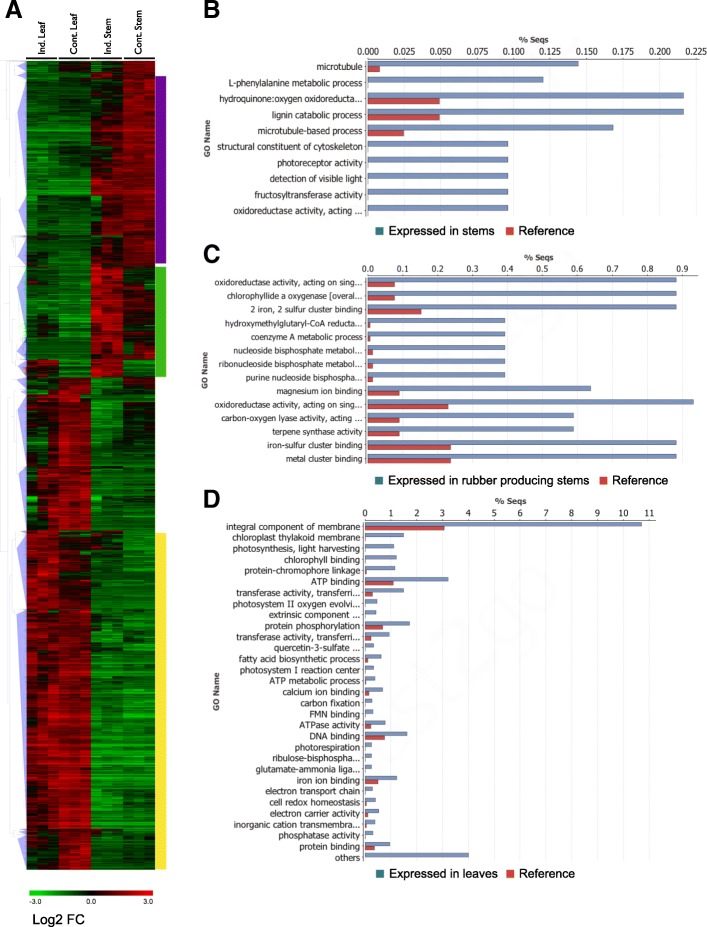
Hierarchical clustering and analysis of differentially expressed contigs **a**) Hierarchical clustering of differentially expressed transcripts. Clusters of contigs with highest expression in specific tissues are highlighted and further examined by GO-term enrichment analysis: stems (purple, **b**), rubber producing, induced stems (green, **c**) and leaves (yellow, **d**). GO terms significantly enriched using in the cluster of contigs most highly expressed in stems (**b**) at a *P* value of < 0.005, in rubber-producing induced stems (**c**) at a P value < 0.0001 and in leaves (**d**) at a P value of < 0.0001 (Fisher’s exact test)

This discrepancy between the number of genes differentially expressed between tissue types and the much smaller number differentially expressed between growth conditions is easily observed in a heat map of hierarchically clustered expression data (Fig. 3a). Hierarchical clustering of differentially expressed contigs shows that most genes exhibit similar expression patterns between tissues in both induced and control plants while a smaller group of genes are specifically expressed in the rubber-producing stems of cold-induced plants (Fig. 3a, green bar). Most contigs in this cluster have a background level of expression in the stems of control plants and are more highly expressed in the stems of induced plants.

### Gene ontology analysis

To characterize changes in gene expression between tissues, we analyzed the gene ontology term (GOterm) enrichment of differentially expressed contigs with similar expression patterns marked with colored bars in Fig. 3a. We analyzed the GO-terms enriched in each cluster of differentially expressed contigs. In the cluster of contigs most highly expressed in stems of control plants we observed the enrichment of cytoskeletal, lignin catabolic, and photoreceptor activities at *p* values below 0.005 (Fig. 3b). In the cluster of contigs highly expressed in the rubber-producing stems of induced plants contigs encoding hydroxymethylglutaryl-CoA reductases, oxidoreductase and terpene synthase activities, which include enzymes families known to be involved in rubber biosynthesis, were enriched at *p* values below 0.001 (Fig. 3c). Nucleoside metabolic processes were also enriched in the rubber biosynthetic gene set. We also analyzed GO term enrichment in contigs highly expressed in leaf tissues (Fig. 3d). As expected, most of the GO-terms enriched in the leaf-expressed gene set were for processes related to photosynthesis such as chlorophyll binding, ATP metabolic processes and electron transport chain at p values below 0.001. Membrane proteins were overrepresented in this gene set, as were enzymatic activities associated with carbon fixation and electron transport chains.

### Analysis of highly expressed and strongly induced genes

To elucidate the metabolic processes occurring in rubber biosynthetic tissues, we analyzed the contigs to identify the most highly expressed (Fragments Per Kilobase of transcript per Million mapped reads (FPKM) > 256) and most strongly enriched (FPKM_Induced Stem_ / FPKM_Control Stem_ > 13) transcripts in induced stem tissues. (Additional file 2: Tables S1 and S2). The most highly-expressed and among the most strongly enriched transcripts in rubber biosynthetic tissue encodes a protein similar to the ribosome-inactivating lectin that is processed into ricin toxin, (TR149020_c3_g1_i1), which is expressed at 7583 FPKM in rubber-producing stems. That this lectin has not previously been identified in proteomic analyses of guayule rubber may be due to the absence of strong homologs in Genbank when previous studies were conducted, or the possibility that this lectin is not bound to guayule rubber particles and is not abundant in preparations of washed rubber particles. Other highly expressed genes are involved in responses to stress and defense responses such as defensins, dehydrins, heat shock proteins (HSPs), metallothionin, COLD-REGULATED 413 and RCI2A. This finding, along with the induction of contigs encoding chitinases implies that the production of rubber in guayule may have a role in defense against herbivory and fungal pathogens as does the production of latex in *Hevea.* Abundant proteins in *Hevea* latex have defense roles; hevein, the primary allergen in *Hevea* latex, constituting 20% of the dry weight of the lutoid fraction, has chitinase and antifungal activity [18]. Hevein functionally agglutinates rubber particles in *Hevea* latex and this activity along with its chitinase and chitin-binding activities are thought to function in protection against insects and fungi.

Other highly expressed contigs in rubber-producing tissue encode proteins with roles in defense, including TR8884_c0_g1_i1, encoding a defensin family protein, and two contigs with homology to MLO6 (Mildew Resistance Locus O 6). Several contigs encoding proteins previously identified as being involved in responses to cold were identified, including contigs encoding homologs of HSPs, COR413-PM2, RCI2A and a dehydrin. A contig encoding a homeobox transcription factor with homology to ATHB7 was also highly expressed. ATHB7 is both cold and drought responsive in Arabidopsis and along with ATHB12 is thought to fine-tune growth during water stress [19].

Analysis of the contigs most strongly induced in stem tissue (Additional file 2: Table S2) identified many additional genes with putative functions in responses to stress and in the production of terpenoids. Numerous HSP-encoding contigs were strongly induced in stems following cold treatment. Several contigs encoding oxygenases were strongly induced, including several with homology to ACD1-like (Pheophorbide-a-oxygenase), a 2-oxoglutarate and Fe(II)-dependent oxygenase-family protein as well as CYP79A2, which catalyzes the monooxygenation of phenylalanine in the biosynthetic pathway for benzylglucosinolate in Arabidopsis [20]. Several strongly induced contigs encode terpene synthase-like enzymes with homology to TPS02 and ATTPS-CIN, which catalyze the production of farnesene from farnesyl diphosphate and diterpenoids from geranyl diphosphate, respectively. The contig TR96894_co_g1_i1 has homology with OLEOSIN1. Oleosins are structural components of oil bodies in plants and affect the size and lipid composition of oil bodies [21, 22], which is relevant given that rubber particles are thought to be structurally related to oil bodies and the small rubber particle protein (SRPP) is homologous to the lipid droplet associated proteins (LDAPs) [23]. Further studies should examine the roles of terpene synthases and oleosin in the biosynthesis of rubber in guayule.

### JA biosynthetic genes

In previous analyses, the most abundant protein found in guayule rubber particles, comprising up to 50% of total protein was found to be a cytochrome p450 enzyme homologous to Allene Oxide Synthase (AOS) [24], a normally plastid-localized protein catalyzing a reaction in the biosynthetic pathway for jasmonic acid (JA), a terpenoid plant hormone. The role of AOS in rubber biosynthesis remains unclear. One homolog of AOS is highly expressed in rubber-producing tissue (Additional file 2: Table S3) while other homologs have distinct expression patterns with highest expression in leaves. Likewise, distinct orthologs of lipoxygenase and allene oxide cyclase, the enzymes that produce allene oxides, are also upregulated in rubber-producing tissue. Homologs of enzymes beyond the allene oxide cyclase step of JA biosynthesis are not significantly upregulated in rubber producing tissue. This result was unexpected and may suggest that AOS has a biochemical role in rubber biosynthesis other than the production of JA.

### Identification of rubber biosynthetic enzymes

To characterize the expression of contigs encoding enzymes previously implicated in the production of rubber in guayule we searched the differentially expressed gene set for contigs encoding the small rubber particle protein (SRPP), rubber associated Cis-prenyltransferases (CPT and CPTL), allene oxide synthase (AOS), DXP and mevalonate pathway enzymes using BLAST, then retrieved annotation and expression data for the identified contigs. Through this analysis we identified a set of contigs encoding AOS, CPTs, SRPP, 3-hydroxy-3-methylglutaryl-coenzyme A reductase (HMGR) and geranyl diphosphate synthase that were generally strongly expressed in induced stem tissue and with lower levels of expression in the stems of control plants (Additional file 2: Table S3A). A consistent background level of expression between one third and one half the absolute transcript level in induced stem tissue was observed in the stems of control plants, in agreement with the qPCR data for selected genes in Additional file 1: Figure S1. The high standard deviation of transcript levels in this study, likely due to the genetic heterogeneity of the polyploid plants sequenced, causes comparisons of expression levels between induced and control stems to be above the stringent threshold used for calling statistical significance. The varying levels of expression of these contigs agree well with qPCR data (Additional file 1: Fig. S1) and previous analyses such as [12]. Each of the contigs identified in this analysis is significantly up-regulated in induced stems compared to expression levels in leaf tissues and is more highly expressed in induced stems than in control stems but not at a significance level of *p* < 0.0001. The weak, but consistent induction of the SRPP-encoding gene and the strong expression of AOS in rubber-producing tissue are consistent with previous studies. The presence of many contigs encoding HMGR may also suggest the presence of multiple alleles or tandem duplication and diversification of this gene in guayule and was also observed in GO term enrichment analysis. A previous EST study identified two distinct HMGR encoding ESTs and did not detect cold induction of HMGR [12] although another study [16] supported that the induced rate of HMGR limits the rate of rubber formation in guayule. Other mevalonate and 1-deoxy-d-xylulose 5-phosphate (DXP) isoprenoid biosynthesis pathway enzymes were identified in the set of differentially expressed transcripts, however all were more strongly expressed in leaves than in stems (Additional file 2: Table S3B) and were not significantly up-regulated in stems following cold induction. Homologs of both classes of rubber-biosynthesis-associated CPTs were detected, homologs of Arabidopsis cis-prenyltransferase 1 (CPT1) and Nogo-B receptor-like CPTL/LEW1 [25]. Notably, the latter is strongly implicated in rubber biosynthesis in Russian dandelion, but homologs are absent in the *Hevea brasiliensis* genome, indicating that the mechanism of rubber biosynthesis may differ between species [26].

### Transcription factors expressed in rubber biosynthetic tissues

**Fig. 4:**
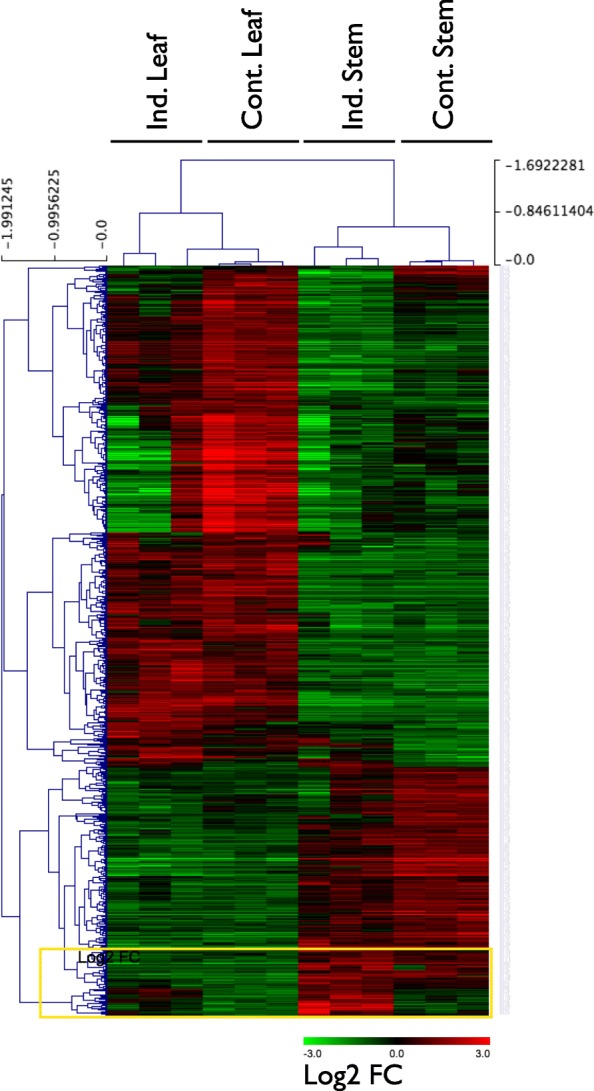
Clustering analysis of transcripts encoding proteins with homology to DNA-binding proteins or transcription factors. Transcripts clustered together and most highly expressed in induced stems are marked by a yellow box and are further examined in Additional file 2: Table S4

To identify transcription factor-encoding genes differentially expressed in rubber biosynthetic tissue we searched for contigs with annotated DNA-binding or transcriptional regulation activity in the GO term dataset. 708 contigs were identified with predicted DNA binding or transcriptional regulation activities. We used hierarchical clustering of the expression of these differentially expressed contigs encoding proteins with DNA-binding or transcriptional regulation activity to identify a cluster with stronger expression in induced stems than in other tissues and at least 2.7-fold higher expression in induced stems than in control stems (Fig. 4, yellow box). This gene set consists of 30 contigs encoding proteins with homology to DNA binding or transcription factors. All but one contig identified through this analysis encode a gene product with homology to previously studied families of transcription factors. TR97361_c0_g6_i1, encodes a homolog of AtGRP2B, which has nucleic acid binding domains and therefore was retrieved in this analysis, but has been shown to act as an RNA chaperone, not a transcription factor [27]. AtGRP2B is a cold-shock domain protein thought to function in the destabilization of RNA secondary structure. Some but not all of these contigs are differentially expressed at *p* < 0.0001 between induced stems and all other tissues and many distinct classes of plant transcription factors are represented in the gene set (Additional file 2: Table S4).

## Discussion

Transcriptome analysis of cold-induced guayle stem resulted in identification of several highly induced genes. The experimental approach using leaves and summer simulated plants as controls allowed elimination of induced genes unlikely to be involved in rubber biosynthesis and its regulation. The identified genes included several that are known or expected to be involved in phenylpropanoid and rubber biosynthesis, thus confirming the validity of our approach. Rubber induction was very highly induced in our plants by the cold treatment (Additional file 1: Figure S1), more than would perhaps be expected by the increased transcripts of known or suspected rubber biosynthetic genes (Additional file 1: Figure S1C and Additional file 2: Table S3). However, the pathway for rubber biosynthesis is not fully understood, and the main rate limiting steps could involve proteins that have not yet been identified. Notably, Additional file 2: Table S2 shows several contigs of proteins with no or limited known functions that highly expressed in induced stems and specifically upregulated in that tissue. These would be good candidates for future studies to validate their possible involvement in rubber biosynthesis. We were particularly interested in transcription factors, since master regulators are ideal targets for genetic engineering of crops. Upregulation of a master regulator will in principle upregulate the entire biosynthetic pathway, and important accessory pathways e.g. for substrate production [28]. Knowing all the biosynthetic enzymes is not necessary for such an approach. In this study we identified several putative guayule transcription factors, which have Arabidopsis homologs with demonstrated roles in the response to abiotic stress or to ABA; ATHB7. DREB1D/CBF4, RD26, AtGSFB2A, AtMYB14, HAT22/ABIG1 AtHSFA2 and AtHSFA6B have been shown to have roles in the response to abiotic stresses or to ABA. These guayule transcription factors are good candidates for regulators of the rubber biosynthetic process given that rubber is produced in a specific tissue, in response to cold. ATHB7 is responsive to drought stress and represses water loss in Arabidopsis [19]. TR42239_c0_g3_i1 and TR42239_c0_g1_i1 are specifically expressed in induced stem tissues and are homologs of DREBA4, a member of the ERF/AP2 family of transcription factors, which are broadly involved in responses to stress and in regulating development. TR78450_c1_g1_i1 and TR78450_c1_g2_i1 encode homologs of DREB1D/CBF4, which is a well-characterized member of the dehydration-responsive element binding and C-repeat binding factor subgroups of ERF/AP2 family transcription factors. Previous studies have shown this subgroup of transcription factors to regulate acclimation to drought and cold stresses [29]. Overexpression of a homolog of CBF4 in poplar induces constitutive tolerance for multiple abiotic stresses including both drought and cold [30]. Thus, overexpression of a CBF4 homolog alone in poplar induced physiological responses associated with acclimation to and conferring tolerance for abiotic stresses. In Arabidopsis, RD26 interacts with BES1/BZR family, brassinosteroid-responsive transcription factors to mediate crosstalk between drought and brassinosteroid signaling [31]. TR91940_c0_g1_11 encodes a homolog of AtMYB14, which mediates cold tolerance in Arabidopsis and was found to negatively regulate freezing tolerance [32]. ABA INSENSITIVE GROWTH (ABIG1) is a stress and ABA regulated transcription factor that functions to restrict growth and promote senescence in Arabidopsis [33]. A guayule homolog of ABIG1 is induced in stems following cold treatment and weakly induced in cold-treated leaves.

## Conclusion

The gene sets identified in this study include strong candidates for master regulators of the cold-responsive, stem-specific biosynthesis of rubber in guayule. Further studies should examine potential uses of these transcription factors for the improvement of rubber yields in guayule.

## Methods

### Plant growth

Guayule (*Parthenium argentatum* Gray) plants of the AZ-2 cultivar were germinated in a greenhouse in Eloy, AZ. At 4 months of age these plants were transferred to simulated summer conditions in a plant growth chamber (16 h day, 25 °C daytime and 15 °C nighttime temperatures). At 6 months of age plants were transferred to a growth chamber simulating winter conditions (11 h night, 25 °C daytime and 5 °C nighttime temperatures) for induction of rubber biosynthesis. Plants were harvested for analysis at 8 months of age. Plants grown in simulated winter conditions were considered “induced” for rubber biosynthesis. Washed rubber particle extractions were conducted as described by [34].

### Quantitative RT-PCR

RNA was extracted from plant tissues using Trizol (Invitrogen) following the manufacturer’s instructions with the exception that following the initial homogenization and incubation in Trizol, insoluble material was pelleted by centrifugation. Following Trizol extraction, RNA samples were treated with DNAase using the Turbo DNAfree kit (Ambion) and further purified using RNeasy column purification (Qiagen). RNA concentration was measured on a Nanodrop spectrophotometer. RNA was reverse transcribed using the Superscript III kit from Invitrogen. Primer sets for qPCR were designed based on *Parthenium argentatum* sequences previously deposited in Genbank as well as EST data from the Compositae genomics project (see Additional file 3). qPCR was conducted on a Stepone Real-Time PCR system (Applied Biosystems) using SYBR® Select Master Mix (Thermo Fisher Scientific) according to the manufacturer’s instructions. qPCR analysis was conducted in biological duplicate with triplicate technical replication.

### RNA seq analysis

Directional, bar-coded illumina sequencing libraries were prepared using the NEBNext® Ultra™ Directional RNA Library Prep Kit for Illumina® (New England Biolabs) according to the manufacturer’s instructions with the suggestions for size selection of an average insert size of 300–450 bp. For library preparation, RNA samples were prepared as for qPCR analysis and analyzed using a Bioanalyzer 2100 RNA chip (Agilent Genomics) to evaluate RNA concentration and quality prior to library preparation. Libraries were analyzed on a Bioanalyzer 2100 High Sensitivity DNA assay, pooled at an equimolar ratio. The pooled libraries were then sequenced on Illumina Miseq (2x300bp) and HiSeq2500 (2x150bp, Rapid Run mode) platforms.

### Transcriptome analysis

RNA seq data was quality controlled and adapter sequences removed using Trim Galore (Babraham Bioinformatics group) to eliminate adapter sequence contamination and to trim data below Q30. The command used was ‘trim_galore --quality 30 --paired --adapter AGATCGGAAGAGCGTCGTGTAGGGAAAGAGTGT --adapter2 AGATCGGAAGAGCACACGTCTGAACTCCAGTCA --length 30 $1 $2’. Miseq and Hiseq reads were pooled for transcriptome assembly. The transcriptome was assembled using Trinity assembler version 2.04 set to minimum kmer coverage of 2 [35]. The command used was “Trinity --seqType fq --max_memory 240G --SS_lib_type RF --min_kmer_cov 2 --CPU 40 –left”. The initial transcriptome assembly was filtered using a FPKM cut-off of 0.5 to eliminate transcript isoforms with low abundance and low coverage. Differential gene expression analysis was performed using the EdgeR as described in [35]. The differentially expressed gene set was selected as contigs with a *P* value, adjusted for the false-discovery rate, below 0.0001.

### Functional annotation

Functional annotation of contigs was performed by searching against NCBI’s non-redundant protein sequence database (NR), the Arabidopsis predicted protein sequence database and the Streptophyte predicted protein database retrieved from UniProt, using BLASTx with default parameters. GO functional categories were assigned to differentially expressed genes by homology using Blast2GO (BioBam, Spain). Hierarchical clustering analysis was performed and visualized using Multiple Experiment Viewer version 4.8 [36]. GO-term enrichment analysis was performed and plotted using Fischer’s exact test implemented in Blast2GO.

## Additional files

Additional file 1: **Figure S1.** Images of induced and control plants analyzed in the RNAseq study. (PDF 547 kb)

Additional file 2: **Tables S1-S4** in Excel format. **Table S1.** The most highly expressed contigs in rubber-producing induced stem tissue. **Table S2.** The most strongly induced contigs in rubber-producing induced stem tissue. **Table S3.** Contigs encoding enzymes that are implicated in the biosynthesis of rubber and isoprenoids and are strongly expressed in rubber biosynthetic tissue. **Table S4.** Transcription factor-encoding contigs most highly expressed in rubber-producing stem tissues. (XLSX 33 kb)

Additional file 3: **Table S5.** Primer sequences used for RT-PCR. (PDF 20 kb)
