# Supplementary figures and images for: Transcriptome analysis of rubber biosynthesis in guayule (Parthenium argentatum gray)
Source: BMC Plant Biol. 2019 Feb 12;19:71. doi: 10.1186/s12870-019-1669-2 (PMC6373111; doi:10.1186/s12870-019-1669-2)

**Control**

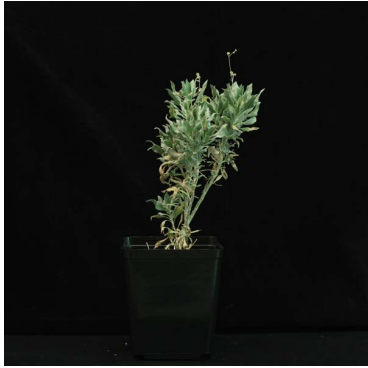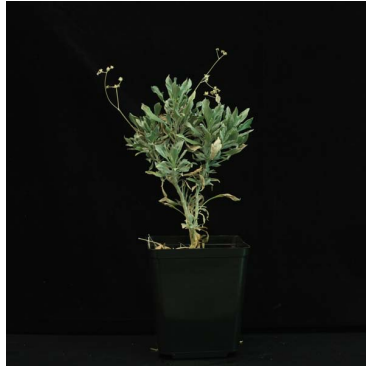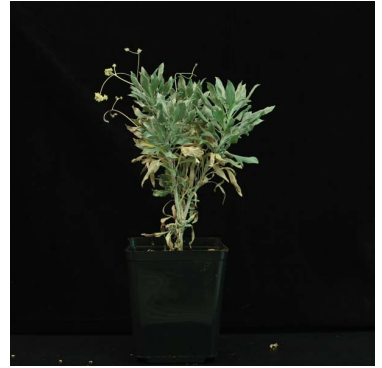

**Induced**

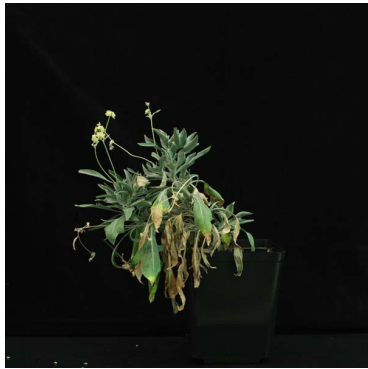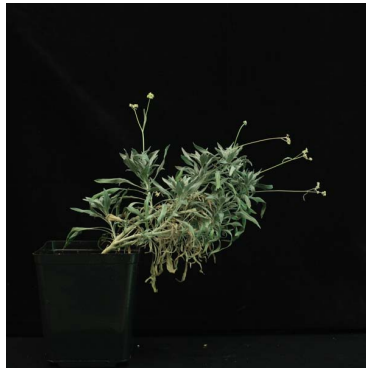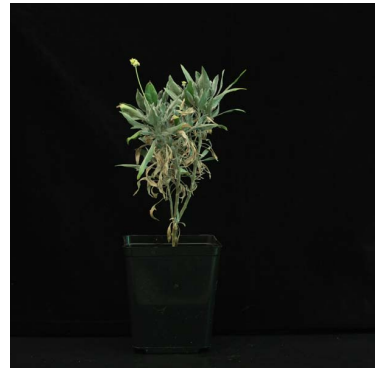

**Figure S1.** Images of induced and control plants analyzed in the RNAseq study.

Supplement: Supplementary file 1 — Figure S1. Images of induced and control plants analyzed in the RNAseq study. (PDF 547 kb) [file 12870_2019_1669_MOESM1_ESM.pdf]
