# Supplementary material for: Transcriptome analysis of rubber biosynthesis in guayule (Parthenium argentatum gray)
Source: BMC Plant Biol. 2019 Feb 12;19:71. doi: 10.1186/s12870-019-1669-2 (PMC6373111; doi:10.1186/s12870-019-1669-2)
Supplement: Supplementary file 3 — Table S5. Primer sequences used for RT-PCR. (PDF 20 kb) [file 12870_2019_1669_MOESM3_ESM.pdf]

Table S5. Primer sequences used for RT-PCR.

AOS F	TTTTATAAGCAGCAACCCAAAAGTCATTGTCCTC
AOS R	ACAAGAGGTTCTTAAGTTGAGCATGTCTAGGTTC
CPT F	GGAGCTCCGGGGAGACGAGA
CPT R	GCCCCATCTCCGGCCACAAC
FPS F	TTACTACTCATTTTATCTTCCAGTTGCCTGTGCA
FPS R	ATGTCTGTTCCAATCTTTCCAATAACCTCAGGAG
SRPP F	AACGCCGGTTCGTGGAAGCC
SRPP R	CGGTCACCAAGTCGCCCACC
UBQ F	CCTTTGTTGCACAGGCGGG
UBQ R	TGGGAGAACACTTGCTGACTACAACA
